# The Synergy Zone: Connecting the Mind, Brain, and Heart for the Ideal Classroom Learning Environment

**DOI:** 10.3390/brainsci13091314

**Published:** 2023-09-13

**Authors:** Janet N. Zadina

**Affiliations:** Brain Research and Instruction, New Orleans, LA 70002, USA; janetzadina@gmail.com

**Keywords:** flow, brain synchronization, heart coherence, education, emotion, learning, classroom environment, engagement, attention

## Abstract

This paper proposes a new perspective on implementing neuroeducation in the classroom. The pandemic exacerbated the mental health issues of faculty and students, creating a mental health crisis that impairs learning. It is important to get our students back in “the zone”, both cognitively and emotionally, by creating an ideal learning environment for capturing our students and keeping them—the Synergy Zone. Research that examines the classroom environment often focuses on the foreground—instructors’ organizational and instructional aspects and content. However, the emotional climate of the classroom affects student well-being. This emotional climate would ideally exhibit the brain states of engagement, attention, connection, and enjoyment by addressing the mind, brain, and heart. This ideal learning environment would be achieved by combining proposed practices derived from three areas of research: flow theory, brain synchronization, and positive emotion with heart engagement. Each of these enhances the desired brain states in a way that the whole is greater than the sum of the individual parts. I call this the Synergy Zone. A limitation of this proposed model is that implementation of some aspects may be challenging, and professional development resources might be needed. This essay presenting this perspective provides the relevant scientific research and the educational implications of implementation.

## 1. Introduction

The pandemic greatly affected students. As a result, several issues affecting students have become critical. Mental health issues are escalating [1]. Teachers report across educational social media that students are lacking focus, have trouble paying attention, and are engaging more frequently in problematic behavior. Social isolation during the pandemic increased in some student populations [2], contributing to depression rates as high as 89% in African American students, for example [1]. As teachers around the world have said to me, “our students are not the same as before”. We must recapture them.

This paper proposes a model for increasing behaviors that mitigate anxiety, and increase focus and attention, engagement, enjoyment, and connection. This model proposes addressing the negative brain states by incorporating practices that create an ideal learning environment. To do that, we must first define what an ideal learning environment would be in terms of practices and brain states. I call this the Synergy Zone.

*“I’m tuned in. I’m getting good vibes. I feel you. We are on the same page. I’m going with the flow. My heart was in it. We are in sync. I was in the zone!*” When people say these things, they are referring to a positive emotional/psychological state that allows them to achieve optimal performance. What does it mean to be “in the zone”? What is that “zone”? How can we elicit it and harness its power in the classroom?

Most people are familiar with the optical illusion that appears to be a vase in the foreground but if you focus on the background, you see two faces. However, you cannot see both at once. It is only by switching your attention to either the foreground or the background that you can see two very different things. Using this concept of foreground/background as a way of viewing the classroom we can consider the foreground to be the aspect that most educators focus on, i.e., the educational strategies, practices, theories, and structure. Of course, considering these is very valuable and essential. However, when focusing on such things as individual students, instructional practices, learning strategies, classroom activities, and assessments, one might miss the background—the psychological/emotional climate of the classroom. The vase—the foreground—is only half the picture.

Research that examines the classroom often focuses on the foreground—instructors’ organizational and instructional aspects [3]. However, the emotional climate of the classroom affects student well-being [4]. As Bandura states, it is the entire person that thinks and feels, not just the brain [5]. 

It is essential that the emotional climate be one that is trauma-sensitive. Addressing student mental health is critical [6]. Recent research on mental health effects indicates that mental health issues such as anxiety, stress, trauma, and depression are on the rise. A study of first-year college students found that moderate–severe anxiety increased by 39.8 percent and moderate–severe depression increased by 47.9 percent from before the start of the pandemic to the culmination of the study in June/July 2020 [1]. A survey of students by YoungMinds found that 83 percent felt that the pandemic worsened pre-existing mental health issues [7].

Education research, while studying groups of students for data collection, usually focuses on how teaching affects individual students [3,8,9]. There has been a movement toward teaching emotional intelligence or social/emotional learning teaching students about their emotions and strategies for regulating them and strategies for interacting with others. This is a very valuable addition, but there is more to student well-being and a learning environment. Rather than focusing on the mental health and well-being aspects of emotional intelligence [10], or social–emotional strategies and instruction focusing on individual behavior, this paper examines the overall cognitive, psychological, and emotional environment conducive to student well-being that reduces anxiety and stress by fostering the brain states of engagement, attention, connection, and enjoyment—qualities counteractive to the brain states of stress, anxiety, and depression.

Let us shift perspective and look at the gestalt—the wholeness, the connections between parts that give it a pattern, the background that may be bigger than the sum of its parts. Totality is the lens through which we are looking. It emerges when our perspective shifts. The lens through which we look at individual students and practices is not the lens through which we look when we see totality. This totality includes student-to-content, student-to-student, student-to-teacher, and student-to-self and world relationships which could be viewed as a joined totality [11,12].

While it is always desirable to focus on individual students and individual practices, one must also switch perspective to seeing students as a collective, as an interactive totality with its own gestalt or atmosphere. We tend to shy away from constructs such as mood, energy, and atmosphere because they cannot be well measured. This gestalt may not be measurable, but it can be observed when perspective shifts. Experienced administrators can walk into a classroom and “feel” the atmosphere, the learning environment, and whether it is positive and likely to encourage learning or whether it is negative and not conducive to learning, before they examine the individual strategies, objectives, lesson plan, or practices going on in that moment. While cutting-edge research using EEG technology in the classroom can measure attention and engagement, we cannot measure the overall gestalt. But just because it cannot be measured does not mean that it does not exist, cannot vary in degree, and cannot underlie outcomes. 

What factors contribute to this ideal background? How can we create an ideal atmosphere in which a group of students is a connected whole with each student being part of this greater whole? How can we make the whole greater than the sum of its parts? Within this environment, potentially every student would be experiencing an atmosphere conducive to academic success and personal well-being while still utilizing individual strengths, skills, and interests. This environment is also ideal for addressing neurodiverse students. While we continue to address their individual needs through our classroom practices and strategies, we also provide an environment in which they are part of the whole. All students have a need for mental well-being. Practices that help students become engaged and focused while feeling safe and connected with lessons that provide for individual skills and strengths can work for all students. Implementing this ideal learning environment is a huge challenge so we must bring together knowledge and practices from multiple fields. What does research coming from education, business, neuroscience, and psychology tell us about the best mental states, emotional states, and classroom practices that can achieve those states? 

This paper proposes a design called the Synergy Zone using the synergistic effects of flow, brain synchrony, and heart coherence to create states of engagement, attention, connection, and enjoyment to get students into the zone of enhanced learning. 

### 1.1. Definitions

The ideal classroom environment would capture the entire student to create a holistic, synergistic effect. At the same time, it incorporates the student as part of a whole, being enhanced by the environment and interactions within it. I am addressing the entire student through the labels of Mind, Brain, and Heart while acknowledging that the boundaries are arbitrary and porous and are also related to brain processes. The section on Mind will address the learners’ engagement with the material or experiences in the classroom, including interactions with others. We will look at top-down strategies initiated by the learner and behaviors in response to the learning tasks and experiences.

The category of Brain will refer to the unconscious synching of the brain waves of the learner to the people involved—peers and instructor. Bottom-up processes will direct the learners’ attention and engagement processes in the brain. Looking at the brain processes can inform educational practices [13].

Heart will refer to both conscious and unconscious, top-down and bottom-up processes and behaviors, that sync the learner to the self, others, and the greater world around them. It includes positive emotion and heart coherence.

### 1.2. The Classroom Climate

What qualities could describe the ideal classroom learning environment that would address the entire student, both as an individual and as a group entity? After an extensive review of the literature, I propose that there are four brain-state components: engagement, attention, connection, and enjoyment. Each plays an important role and together they create a synergistic effect wherein the whole is greater than the sum of its parts.

I further propose that focusing on practices derived from three areas of research can create a learning environment that puts students in a synergistic zone of engagement, attention, connection, and enjoyment. These research areas are flow, brain synchronization, and heart engagement. While the concept of flow has been around since the early 1990s [14], the concepts of brain synchronization and heart coherence in the classroom are newer. It is time to bring them to the forefront so that current educational practices and strategies can be more effective.

Activating the states of flow, synchronization, and heart engagement creates these brain states, resulting in a “synergy zone”. For example, engaging the mind with the content material and others can create flow which creates engagement and enjoyment. Engaging the brain in synchronization with others creates engagement, attention, and connection and stimulates enjoyment. Engaging the heart with self, others, and life creates enjoyment which reduces stress, which in turn improves attention and creates a connection to the school and to others.

Let us define the desired states and their role in learning, and then the mind, brain, and heart modalities by which we could achieve these states.

### 1.3. Engagement

Engagement is essential to learning [3,15,16,17,18,19,20,21,22,23]. Engagement can be defined as “a psychological process, specifically, the attention, interest, investment, and effort students expend in the work of learning.” [24] (pp. 54–55). Engagement can reduce anxiety by reducing the activation of the default mode network involved in rumination and anxiety [25]. Engagement increases enjoyment [26].

### 1.4. Attention

Attention is critical to learning [3,27,28]. It affects how the brain processes information [29,30] and increases retention [31]. Attention increases enjoyment [26]. The ideal environment must include the focused attention of students.

### 1.5. Connection

The classroom emotional climate has been defined as including relationships [3,32,33,34]. The relationship between teacher and student is a factor in learning outcomes [35,36,37,38]. Students should feel a connection to other students, the teacher, and the school—bringing the heart into the equation.

### 1.6. Enjoyment

The emotional climate of an ideal classroom must, of course, be a positive emotional environment. Recent research indicates that the state of positive emotion—enjoyment— enhances learning [3]. Furthermore, positive emotions such as enjoyment reduce the stress that impairs learning, which is essential for effective learning.

How can we achieve these desired brain states? I propose the approach of synching minds, brains, and hearts to content, others, and self for mental/emotional well-being and achievement. Practices derived from research on flow, brain synchronization, and heart engagement can help us find the way.

## 2. Synching the Mind: Creating Flow to Connect to Content and Others

Our first goal is to engage students’ minds. We need a state of mind that increases engagement. One such state of mind is defined as “flow”. Flow enhances engagement, which supports academic success [39]. Flow can be a characteristic of group interaction as well, thus contributing to the student as part of a whole.

### 2.1. Engagement

An ideal classroom environment includes engagement. Students who are engaged demonstrate greater participation, motivation, and effort [24,40], while disengaged students can be disruptive, have lower achievement, and demonstrate boredom [41,42]. Students were more engaged in classrooms rated high in emotional climate [3,24,43,44] and had better academic outcomes [17,18,40,45,46,47,48], perhaps because they were engaged [3] (Reyes 2012). In fact, it is often described as feeling “in the zone” [14]. In a study examining the relationship between the emotional climate and academic outcomes, Reyes’ lab determined that engagement was the mediating factor controlling for school, teacher, student, and other classroom factors [3]. We can increase the brain’s state of engagement by inducing a state of “flow”.

### 2.2. Flow

Csikszentmihalyi introduced the concept of flow as the optimal psychological experience defined as a state of mind in which most or all the following are present:

- Absorbing with lack of distraction;
- Loss of sense of time passing;
- Diminished awareness of self; the merging of action and awareness;
- Enjoyable [14,49].

This is a state that facilitates engagement with the task, material, or others. Csikszentmihalyi postulates that the following conditions must be present to enhance flow experiences in an individual [14]:

- The activity is freely chosen;
- There is a sense of control;
- Transitions between actions are smooth;
- The task is meaningful;
- The goals are clear;
- Feedback is immediate and concrete.

There is a balance between challenge and skill—between anxiety and boredom [14].

Student satisfaction and attitudes were most affected by the perceived balance of challenge, skill, and feedback [50,51,52], which are important conditions of flow [53,54].

### 2.3. Implications for Education

Flow is not commonly addressed in education [55,56] although a few researchers are studying it in the classroom {Raettig, 2018 #112} [57,58]. This is unfortunate because newer research indicates that a state of flow is associated with academic success [56,59,60,61,62,63,64,65]. Furthermore, a state of flow enhances students’ perceptions of learning, skill development, and satisfaction [50] and helps them persevere [66] The most important aspect of flow that affects learning outcomes is the intrinsic reward inherent in flow—the activity itself is rewarding [50]. Helping students identify their strengths and ways to use them can increase intrinsic motivation [67].

Many engaging activities create flow in individuals. What creates it in the classroom? To increase engagement by creating a classroom environment conducive to flow, we must incorporate practices and tasks that make the condition of flow likely. The three factors most predictive of flow are control, challenge, and attention. [68]. Creating interest in students is critical for flow [21]. Reading creates a flow experience for many individuals [57,69]. A recent study shows a relationship between engagement and how teachers create interactions [3]. Collaborative learning done well can increase positive interactions. Walker adds that to achieve flow in group work, the rules must be clearly defined, followed, and respected by all [70].

Autonomy within the context of rules and responsibilities enhances flow [54,66,71,72]. Educational research shows a positive association between achievement and autonomy [73]. Classrooms supporting autonomy predicted student engagement [74]. Another aspect of control and autonomy is self-efficacy. Flow occurs in activities in which participants experience self-efficacy, an ability to handle the demands of the activity while not being bored by them [49]. As students become more skilled, the complexity level increases, providing them with the proper stimulation and difficulty to create flow. Therefore, an ideal environment must also contain appropriate challenges. Neurodiversity must be taken into account when creating appropriate challenges.

In his research on flow activities, one of Csikszentmihalyi’s most important findings across activities was that they gave participants “a sense of discovery, exploration, problem solution—in other words, a feeling of novelty and challenge” [49] (p. 30). Challenge is a necessary requirement for flow [26,64]. Leisman suggests that it is the complexity, not the difficulty, that creates the appropriate challenge; the thought processes, not the effort [75].

Connection is another component of the ideal environment. The classroom is a social environment; therefore, we want to consider both individual and social flow. Recently, researchers have looked at flow at the group level and proposed that the most effective teaching situation is students working together in interactive flow [21]. The experience of flow was positively correlated with group competence, contributions, and learning outcomes [21]. Educators can design learning experiences that incorporate the conditions for flow in groups. In a group, it is the interaction that creates flow [21]. Walker [70] asks whether social flow is the sum of its parts—the flow experiences of the individuals added together—or if it could create a phenomenon that is greater than the sum of its parts. Walker speculates that “people may serve as agents of flow for each other” [70] (p. 4). Could the gestalt be greater than the individual effects of lesson designs? The challenge for educators is to design lessons in a way that group members can achieve flow.

Flow works synergistically by increasing enjoyment and engagement. Overcoming challenges elicits positive emotions. According to Chaouachi’s research using EEG to measure brain states, positive emotions result in the highest level of learner engagement [16]. Creating an opportunity for learners to experience flow would enhance the learning experience and improve outcomes by creating enjoyment [70] and engagement [21].

Can we create a process in the classroom that leads to greater engagement such as choice/control + challenge + collaborative learning = flow > increases enjoyment, connection, and engagement. I am not implying that there is a direct, linear relationship because each aspect is interrelated and complex, but creating a “formula” of sorts helps to guide our approach.

We have seen that engagement is essential and that we can create it by engaging the mind to the content and others through flow. This state of engagement can be predicted by brain synchrony [15], leading to the second aspect of the connected classroom—the connected brain.

## 3. Synching the Brain: Creating Synchronization to Connect to Peers and Instructors

We have set the stage for increased engagement with content and others by creating opportunities for flow. But to benefit from this design, the learner must be “in sync” to be in the classroom group flow. This requires attention. Brain synchronization cannot occur without attention.

### 3.1. Attention

Attention in the classroom is often top-down, consciously directed, involving theta waves which, as seen via EEG, are affected by task engagement [76,77,78]. Prolonged engagement increases children’s theta power [77]. These top-down attentional processes are critical to learning [77]. Attention can also be bottom-up, resulting from a strong external stimulus [77,79].

In prior educational research, attention was measured by behavior via observation or self-report. However, observing student behavior does not always give an accurate representation of attention [80]. For example, students looking away during group work might reflect engagement rather than disengagement [81,82]. Some other behaviors assumed to reflect a lack of engagement might be microflow, so this is not always reliable [49] (xxv). Self-reporting is also not ideal. Measuring engagement in neurodiverse students has been problematic [83], but is now measurable.

New technology allows us to measure engagement by looking at physiological measures [84]. NASA developed an index to measure engagement for pilot safety in flying [85]. In subsequent research, brain synchrony measurements via EEG reflected engagement [9,84]. Although there has been little research on the underlying brain processes of engagement and attention in educational settings [12,86], recently developed portable EEG technology now allows us to measure brain waves in a classroom setting rather than under artificial conditions in a laboratory [12,15,87,88,89,90,91,92,93,94]. These brain wave measurements reflect levels of attention.

Researchers found that brain waves could synchronize between students and between instructors and students [15]. Synchronization with a stimulus depends upon attention [30,95]. When people are engaged in joint attention, their behavior begins to synchronize [15,86,92,96,97]. Understanding and enhancing this synchronization can benefit attention and connection.

### 3.2. Brain Synchronization

Self-synchrony is implicit in normal behavior as our body and brain are intricately related [98]. As early as 1966, William S. Condon examined the concept of synchrony at higher-order levels [98]. Using a time–motion analyzer, a polygraph, and film, he slowed down films of speakers and his microanalysis showed self-synchrony—the speaker’s body movements were synchronized with his speech segments [98], or as he put it, “the body dances in time with speech” (p. 255). Condon says this applies to all humans although cultural differences might reflect a different rhythm.

Condon made what he calls a “startling” discovery at the time: interactional synchrony between speaker and listener, with the listener moving to the speech as the speaker does. This happened consistently during conversations except when one was removed and widely separated. He states that “it is as if the listener’s whole body were dancing in precise and fluid accompaniment to the speech” (p. 306). This happens when the listener is paying attention and able to move. In fact, he states that this interactional synchrony starts at birth and perhaps in utero and may be important to language acquisition. Behavioral synchrony has been validated in later studies [38,99].

Individuals in pairs or groups, including teacher–student pairs, often imitate each other through behavioral contagion [35,100] and the extent of this behavioral synchrony is related to student comprehension [101,102]. In 1988, also using film, behavioral synchrony was observed in high school students, with the degree correlated with rapport ratings between students [38]. Synchrony is essential to group collaboration [103,104] and can predict learning outcomes [105].

We can observe behaviors synchronize, but what about brains? Condon speculated that speaking and listening might use the “same rhythmic organizing processes of the brain [98] (p. 309). As it turns out, he was prescient. Synchrony in mind and movement has been substantiated in more recent research using physiological measures [27,96,106]. Physiological or “brain synchrony” occurs when physiological measurements between pairs or groups are similar (for a review, see [107]). Using functional near-infrared spectroscopy (fNIRS) and Socratic dialog between teacher and student, “students and teachers dance at the same pace” [13].

Now, with more advanced technology, we can see cognitive states as they occur over a period of time [94,104,108,109]. The most reliable measure of cognitive states is the electroencephalogram (EEG) [16]. The EEG allows us to measure brain synchrony between individuals since it reflects underlying mental processes [12].

EEG technology allows us to measure brain processes during learning and can measure attention and engagement over 16 hours. [85,92,109,110,111,112,113,114,115,116]. The level of synchrony reflects the level of attentional engagement [31,93]. By contrast, lack of engagement was reflected in a lack of synchrony [86].

While brain physiological measurements have primarily been studied in individuals, more recent research examines signals across groups, showing that under some conditions, EEG measurements can synchronize between members of a group engaged in the same activity together [92,117,118]. Synchrony has been shown to occur among students and between students and teachers [9,12,119,120]. In fact, “classroom engagement and neural coherence do go hand in hand” [91]. When all students are paying attention to the teacher’s voice, for example, “stimulus entrainment” occurs [121].

Physiological synchrony is seen in tasks requiring joint attention [30,86,97,106,118,121,122,123]. In fact, joint attention may be essential for synchrony [15]. A study by Stuldreher et al. [124] found that EEG synchrony measurements during auditory tasks reflected the attentional focus of participants, with it being stronger when given the same instructions. This joint attention may be strongly related to connectedness between people [125]. It may be the shared attention that underlies the synchrony [15,86]. Prior eye contact before class could facilitate joint attention [91,126] and may explain why when two people coordinate their attention there is higher interbrain synchrony [118,127].

### 3.3. Implications for Education

Synchrony enhances learning as shown in measurements of communication [84,128], comprehension [84,128], and memory formation and retention [9,27,31,93,119,123,129,130]. EEG measurements predicted performance on posttests on the content, both immediately and with delay [31,93,119], and discerned in the moment between what was remembered or forgotten [120]. Interestingly, the degree of synchronization between the instructor and the learner predicted learning outcomes in some studies [12,131,132]. When students demonstrated learning by performing better on a posttest than on a pretest, synchrony was higher [119].

Stimuli properties affect attention and synchronization, including the teaching style, audiovisual materials, and social interaction [12,97]. Student–teacher synchrony increases if the teacher is the stimulus [12]. Stronger student-to-group synchrony positively correlated to student ratings of teaching style. Synchrony was higher with videos than with lectures [12,86]. Movies elicit brain synchrony relevant to the viewer’s attentional engagement [12,86,97]. Video was preferred over lecture but there was a smaller difference if the teacher was more highly rated by the students as the lecture involved the teacher [15]. However, student focus predicted synchrony levels independent of teaching style. The more focused a student is, the higher the synchrony [15]. Focus is holding attention.

What classroom practices are shown to promote higher synchrony based on measures of attention? Attention was lower in teacher-initiated activities (lecture and video watching) than in student-initiated activities (group and independent work) [81]. Additionally, lectures rated higher than video watching presumably because videos have more opportunity for disengagement. Researchers also found that measurements of attention were higher during the second half of lessons perhaps because as they progressed, students became more engaged. Shared attention affects memory retention [97].

Group dynamics are critical for learning [15,133,134,135]. Most research, however, looks at learning at the individual level [8]. Social interaction is not well-studied in educational research or in neuroscience [9], although some recent studies have shown its relevance to academic performance [117,118,134,135]. A recent study showed that when participants cooperated to complete a task, their brains synchronized; if they made eye contact, the synchronization was stronger [136].

One important aspect of social interaction is the teacher–student relationship. Teacher–student relationships affect achievement [12,43]. Synchrony between a speaker and listener is related to the rapport between them [137]. In a study investigating student–teacher interactions, feeling close to the teacher was reflected in higher teacher–student brain synchrony [12].

Students whose brains synchronized more closely to the brains of classmates and instructors learned better than those without the synchrony. In fact, when students listened to a lecture, the amount of synchrony at a given time in the lecture predicted which questions the student would answer correctly [138]. The correctness of the answers could not be determined by looking at an individual student’s brainwaves but only by looking at the connection—the synchrony—between students and teachers.

Student-to-student interaction plays an important role, especially in group settings. Social affiliation, or connection, is seen in brain responses [139,140]. Synchrony has been documented during social interaction and group affiliation [99,100,141,142]. Synchronization in movement between people increased their sense of affiliation [38,137,141], thus increasing connection, another factor in the Synchrony Zone. In fact, teaching style was not as important as group affinity in student-to-group synchrony [15]. If instructional practices increase group affinity, this could increase student-to-group brain synchronization. Synchrony was positively associated with better learning during collaborative tasks; successful group collaboration involves not only simultaneously working on the same activity, but also being “in sync” [104,143,144].

It has been shown that synchrony is stronger when the task is more difficult [104,142]. It may be that there is less engagement and attention if the task is too easy, and that challenge evokes synchrony [104]. Confusion during tasks may be part of the creative process as long as the mismatch between the demands of the challenge and skill levels is not too great [145]. This is similar to the challenge requirements for flow, emphasizing the importance of creating appropriate challenge levels and illustrating how one component of this model enhances another.

Creating opportunities for synchronization when designing activities can increase engagement, attention, and connection. This concept needs to be introduced through professional development to foster the Synchrony Zone. Aspects of this model work in a synergistic way with the other aspects, potentially creating a whole greater than the sum of its parts. Flow increased engagement. Students who said they were more engaged showed more synchronization with other students [15]. More synchronization led to more attention and more connection. More attention led to more synchronization and better academic outcomes creating a recursive, synergistic effect.

Is it possible to create a process in the classroom environment that increases the important learning components of attention, such as instructional practices > increased connection > increased brain synchrony > increased joint attention = better outcomes?

However, an ideal classroom environment must include positive emotions. It has been widely established since Damasio’s work [146] that learning and emotions are inseparably entwined. As a result, emotions affect learning [3,16,147,148,149]. Current popular thought attributes emotions to brain processes, but newer research reveals that the body’s organs and most significantly, the heart, send information to the brain [150]. The heart affects both emotional and cognitive processing in the brain. To teach the entire student, the heart and the emotional relationship to oneself, others, and the outside world must be addressed.

## 4. The Connected Heart: Creating Positive Emotions and Heart Coherence to Connect to Self, Others, and Life

Emotions are critical to thinking and learning. In fact, the most important cognitive and social/emotional functions required to be successful in school are significantly affected by emotions [151]. Emotional regulation is critical to success in school and in life [152]. Going back to our opening vase illusion, education has historically focused on the foreground, cognitive skills, with inadequate focus on the background aspect of emotions [152]. The heart (emotions) must be an important part of our ideal learning environment, as it underlies the cognitive and social skills required for flow and synchronization and is essential to our academic goals in education.

One aspect of emotions is the ability to self-regulate. Self-regulation is critical to life/school success. Unfortunately, it is negatively impacted by high stress. Educators around the world have reported on educational social media and to me in person that they have seen a large increase in behavioral problems when students returned to class after the pandemic lockdown. Students (and faculty) have been having trouble regulating their emotions.

Stress, anxiety, and depression impair learning in many ways, including the ability to self-regulate [153,154]. One mechanism is through reduced efficacy of frontal lobe executive functions, such as regulation of self, emotions, attention, and the ability to organize, plan, and perform higher-order thinking [152]. The other mechanism is through increased activation of the emotional areas of the brain which can make attention and engagement difficult and lead to increased behavioral problems in the classroom that can disrupt learning. Due to the emotional and cognitive effects of many anxieties and traumas students experience, as well as the effects of the pandemic, the most important factor in creating the Synergy Zone would be to reduce these negative effects.

Many strategies have been proposed recently to reduce these effects, such as breathing techniques and meditation. These are effective and only take a few minutes. However, some classroom practices increase stress so that the effects could resume momentarily. What can be done that might create an overall environment that reduces these effects as well as the creation of stress? One option is to reduce anxiety by increasing the positive brain states of enjoyment, connection, and engagement. Through these brain states, we can create an ideal classroom climate for learning. Increasing positive emotions and connection in the classroom is the third aspect of creating the Synergy Zone in the classroom.

### 4.1. Enjoyment

Enjoyment is a positive emotion that is an important part of the Synergy Zone. Enjoyment enhances learning [57,155,156]. Classrooms that had a positive emotional climate demonstrated enjoyment [157].

Positive emotions improve learning [158,159], self-regulation, resilience, and motivation [160,161,162,163,164,165]. McCraty and Childre found that self-generating positive emotions brought long-term improvements in students’ ability to self-regulate [165]. Their conscious attempts to increase positive emotions led to improvement in the unconscious feelings and behaviors, leading to a rewiring of the negative patterns that had started forming during the pandemic toward pathways of positivity and “more positive emotions, attitudes, and behaviors in daily life” (p. 243). Bargh and Williams suggest that nonconscious emotional regulation could be more effective than deliberate emotional regulation [166].

One way to reduce stress is by increasing positive emotions such as enjoyment. When enjoyment goes up, anxiety goes down [167,168]. The first step is to understand the nature of enjoyment. Enjoyment is a qualitative factor, not a quantitative one, for the most part in actual practice. Enjoyment is like pleasure but not the same as “fun”. Increasing pleasure and enjoyment is not trying to “dumb down”. We do not enjoy working on an easy 10-piece jigsaw puzzle, so there is an element of challenge, which we are creating through flow and brain synchronization. Again, all our components work together to enhance each other.

Emotions have long been associated with the heart. Emotions affect the heart’s rhythms. It is also well-established that human emotions are contagious. This is commonly attributed to mirror neurons causing us to perceive and then feel the emotions of another. There is another mechanism. The heart emits electromagnetic waves that can produce physiological effects in others up to five feet away [165]. Heart rhythms affect not only the individual but also others in the classroom. Activating learners’ hearts in a positive way can be a third way of getting students into the Synergy Zone.

### 4.2. Heart Engagement

Emotions affect heart activity. As measured by electrocardiograms (ECG), heart rate variability (HRV) is a measure of time between heartbeats and enables us to see the effect of emotion on the heart [169]. It reflects the interaction between the heart and the brain. Measurements of HRV can distinguish whether the sympathetic (fight/flight) branch or the parasympathetic (rest and digest) branch is regulating the heart rate.

Emotions affect the autonomic activity which affects the heart’s rhythm, which can be in normal, entrainment, or coherent mode [170]. Negative emotions and anxiety are reflected in erratic and disordered heart rhythms (less synchronization/interaction between the two branches of the nervous system—sympathetic and parasympathetic), i.e., incoherent state. Feelings of frustration create a disordered HRV pattern and increase the flight/flight response. This disrupts the flow of information to the brain, affecting attention, memory, and higher-order thinking [171].

The rhythm is coherent when the heart, brain, and autonomic nervous system are synchronized [150]. Heart coherence refers to the physiology of sustained heartfelt positive emotions [169]. Positive emotions, such as appreciation, love, or compassion are reflected in highly ordered (coherent) patterns in heart rhythm, indicating more synchronization between the two branches [165,169,172]. When the heart is coherent, it transmits signals to the brain that facilitate cognition and emotional regulation [173].

### 4.3. Implications for Education

Have you ever said to someone “it didn’t seem that you put your heart into it?” How can we engage the heart in classrooms? How can we facilitate students’ ability to be in entrainment mode or to have coherent heart patterns? Research on heart coherence reveals that feelings of appreciation, love, and care affect the heart. [174]. Feelings of appreciation decrease sympathetic (fight/flight) activity while increasing the calming parasympathetic system [170].

Students who are experiencing positive emotions are more likely to have coherent heart rhythms. When athletes are in this state, they describe it as “being in the zone”, and it is associated with high enjoyment and better performance [175]. In a study of high school seniors, those who received three weeks’ training in a heart coherence method had significant improvement in test scores and passing rates on mandatory state tests of math and reading [176]. They also demonstrated reduced depression and hostility. High school students had an average gain of 14% in reading scores after only three weeks of training in a method called HeartSmarts designed to activate these feelings in students, along with teaching them about emotions and giving them social/emotional skills [176]. The goal is to find a way to increase feelings of appreciation (gratitude), love, and care in the classroom.

McCraty and Childre devised a technique for focusing attention on the heart area while also focusing the mind on appreciation as a means of inducing gratitude and heart coherence [165]. The Institute of HeartMath has developed tools and strategies for inducing coherence [177,178]. These strategies have been used at all education levels from elementary to graduate levels, improving emotional regulation and academic performance [152,153,154,179,180]. In addition, the relaxation practice of progressive muscle relaxation can increase feelings of love and appreciation [181]. This training could have significant implications for education in terms of both achievement and wellness [51,159].

Do our current structures and practices in education create enjoyment for teachers or students? In research studying award-winning instructors, the most joyful experiences reported were “vigorous, engaging classroom discussions” [182]. Teacher and student enjoyment were found to have a reciprocal effect [183]. How can we incorporate techniques into our teaching methods that increase enjoyment, joy, compassion, connection, appreciation, care, and love?

### 4.4. Increasing Positive Emotions by Activating the Reward Pathway

Positive emotions are pleasurable, which increases enjoyment. Pleasure activates the reward pathway in the brain releasing dopamine, the motivating neurotransmitter. This pathway is also known as the survival pathway [184]. When something is deemed important or pleasurable to the brain, it is more likely to be remembered for survival. Activities that are rewarding (pleasurable) are more likely to be engaged in again.

Learning has been shown to activate the reward pathway [185]. The learner becomes intrinsically motivated via the learning process [49,186]. While both extrinsic and intrinsic rewards can activate this pathway, some extrinsic rewards may not have the same motivating nature in the classroom. When designing lessons, finding ways to activate the intrinsic reward system would be more beneficial than offering extrinsic rewards for accomplishment. The dopamine “rush” from an “aha” moment is one type of intrinsic reward. Incorporating projects that serve others in the class or community offers both extrinsic and intrinsic rewards because a sense of service or connectedness increases enjoyment. These could be built into lesson design. Feeling that an activity is rewarding or pleasurable in itself, “the intrinsic dimension, can be a powerful source of motivation, either alone or in conjunction with external rewards” [49] (p. 22).

Again, our components work together to create a Synergy Zone. A state of flow is self-motivating [187] and intrinsically rewarding [50] so we can increase reward through both flow and heart engagement.

### 4.5. Increasing Positive Emotions through Gratitude (Appreciation)

Feelings of gratitude, appreciation, love, and care are enjoyable emotions, [165] that enhance another aspect of the synergistic classroom: enjoyment. Creating a heartfelt environment with attitudes of appreciation/gratitude can significantly alter the gestalt— climate, mood, and energy—of the classroom in a way that reduces stress and engages the hearts, minds, and brains of students [188].

Gratitude is one of the most important emotions in human experience [33]. Gratitude is a feeling of thankfulness or appreciation for benefits received [189] or benefit triggered (grateful to), or a generalized gratitude for gifts or blessings in life (grateful for) [190] Gratitude research is relatively new (starting with Emmons and McCollough in 2003 [191]). However, there is a growing body of rigorous, controlled research on the science of gratitude, not only with regard to overall well-being and resilience but also learning (for a review see [188]).

Gratitude practices change what one pays attention to, shifting attention from the natural negativity bias to things that make us feel positive. Research interventions with compassion or gratitude training changed activation in brain regions associated with emotion [163,192,193,194]. The practice of gratitude has been shown to reduce stress [191,195,196,197,198] and be protective against trauma symptoms [199]. The act of appreciation increased parasympathetic activity (the rest and digest branch of the nervous system). In a study of priming with resentment versus gratitude, heart rates were lower in those who were subjected to the gratitude intervention [163].

An “attitude of gratitude” has been shown to have numerous other benefits: increased positive affect [191,200,201,202], increased prosocial motivation [199,203], improved ability to cope with natural disasters or violent incidents, better self-regulation [165], more engaged thinking [204], improved resilience and persistence [205,206], increased goal contagion or goal attainment [33,198], higher motivation [207], improved relationships [152,208,209,210], higher subjective well-being [67,191,198,201,211,212], better rapport among undergraduates [208], more cooperative behavior [213], increased social cohesion [214,215], and improved self-esteem [196,212,216,217,218]. Gratitude improved satisfaction with the school experience [201], which is related to academic success [219].

Gratitude can be learned. Newer research investigates gratitude interventions in educational settings [158,201,207]. Educators can find multiple methods for evoking gratitude in students, including daily or weekly gratitude lists or journaling [191,207], counting blessings [201], writing letters of gratitude, [67,200], or taking a moment to choose gratitude over resentment in approaching lessons [204].

Gratitude clearly contributes to the Synergy Zone through increased engagement and enhanced connection to self and others, along with other benefits. Gratitude (appreciation) increases heart coherence which leads to a feeling of connectedness, which in turn enhances group work and fosters brain synchrony. And the greater the brain synchrony, the greater the rapport and sense of belonging [98]. Again, our components enhance each other, creating the Synergy Zone.

## 5. Implementing The Synchrony Zone

Each component has a strengthening effect on the other components with the effect that the total is indeed greater than the sum of its parts. Making a small change in one component can bring the other components into play without focusing on them individually. Each time you incorporate one component you are strengthening “the zone”.

We begin the creation of the Synergy Zone by designing learning conditions conducive to flow. Three conditions associated with flow engage all three important aspects of the ideal learning environment: the balance between challenge and skills engages the mind; focused attention engages the brain; and the positive emotion of enjoyment engages the heart.

While the concepts of flow, engagement, brain synchrony, attention, heart synchrony, and emotion are all intertwined, a simple model to ease application could be that emotion enhances synchrony, attention, and engagement; attention enhances synchrony, attention, and engagement; and flow enhances engagement via immersion in appropriate content, brain synchrony through joint attention, and emotion through the pleasure of flow. By choosing practices that enhance engagement, attention, connection, and emotion we can achieve an ideal classroom environment. The specific teaching methods may not be as much of a significant variable in outcomes if the appropriate physiological states are activated. We have seen that even a lecture format can be as viable as other means when the student has positive emotions (likes the teacher, which presumes enjoyment, attention, and engagement). This is not to say that the instructor should ever strive to be liked. Being liked may result from the classroom environment the instructor created that contains optimal psychological states such as flow (engagement), brain synchronization (attention), and heart synchronization (positive emotions and enjoyment).

**Educator Intervenes Here****Student Engages Here****Ideal Classroom Environment****Mind**FlowContentEngagement, Enjoyment**Brain**Brain synchronyInstructor, PeersAttention, Connection**Heart**Heart engagementSelf, lifeAttention, Enjoyment, Connection

## 6. Limitations

Unfortunately, after over two decades of discussion of “brain-based learning”, “educational neuroscience”, or “neuroeducation”, a bridge between neuroscience and educational practices has still not been adequately implemented, although many developments in cognitive neuroscience and other fields have led to discoveries with implications for improving education [75]. Without an overarching mission and plan for a concerted effort to include certified practitioners of neuroeducation throughout the entire educational system, K–graduate level, concepts such as this and others will be implemented only in limited ways. This lack of effective articulation between lab and school is one major limitation.

There has been a grassroots movement among educators with an intense interest in the brain and in bridging neuroscience and education, leading to conferences such as Learning and the Brain where educators can participate in professional development activities on this topic. Recently, many workshops and programs have become available. However, expertise and content can vary widely. The credibility of the presenter can be an issue. Few creators of these activities have experience and credentials in both education and neuroscience. Therefore, programs to certify instructors are needed.

However, in the meantime, educators worldwide are educating themselves on improved methods for teaching and learning based on science. This essay is designed to provide such a resource.

Another limitation is that creating the Synergy Zone is not a quick fix or something that can be accomplished just by reading this. While reading this will open educators’ eyes to aspects of the classroom environment they may not have previously considered, there will be a learning curve for educators attempting to implement this model. Implementing practices from the brain synchronization and heart engagement sections is fairly easy and implementable in any classroom. However, creating flow is more challenging.

Creating appropriate challenge levels as a component of flow would be the greatest difficulty for most classroom teachers. It means that instruction must be individualized either at the group or within the group levels. With the current recognition of the importance of addressing neurodiversity in the classroom, this imperative should already be in place or being considered at the classroom level. However, having implemented it myself at various grade levels with no help from software, I know it can be done to some degree at the classroom teacher level with enough knowledge and effort. Having said that, schools need to look at software and materials conducive to the adjustment of challenge levels within the classroom. Abundant material is available online and in materials but needs to be organized and accessible.

Professional development needs to be available on the Synergy Zone to help with its implementation. I have seen little in the field of professional development on flow (an optimal psychological state linked to achievement) and no professional development on brain synchronization. Professional development on heart engagement is available, especially through HeartMath, as described above, but not widely prevalent in my experience.

## 7. Conclusions

Our overarching goal is to create an ideal learning environment characterized by engagement, attention, connection, and enjoyment through practices derived from three areas of research: flow, brain synchronization, and heart engagement. I call this the Synergy Zone because each of these three areas has a synergistic effect, with practices from one area affecting the other areas and working together to enhance the ideal brain states for learning.

### 7.1. Flow for Engagement Also Enhances Enjoyment

While flow was discussed here as a means of engagement, it also enhances enjoyment. Enjoyment is part of the flow state by definition [14]. Chaouachi emphasizes that if instructors wish to influence engagement levels, they need to be aware of the strong influence of the learner’s emotional state [16]. Athletic trainers, politicians, factory work designers, art museums, and even police departments have adjusted procedures to increase flow, and thus increase enjoyment [220].

An important component of flow is challenge, and this also enhances enjoyment. “The relationship between challenges and skills is one of the fundamental characteristics of an enjoyable activity” [49] (p. xvi). We have already built that in by providing flow. Positive emotions appear to be a consequence of the flow state [60,64,187] and are related to enjoyment [14,62,70,221]. In a series of three experiments, Walker concluded that social flow is more enjoyable than solitary flow [70]. Creating flow within group learning activities raises enjoyment, leading perhaps to more teacher enjoyment and a joyful connection between the two in the classroom. Emotional contagion effects may be at work [222]. We have shown that the connection between teacher and student enhances brain synchronization. Challenge is a factor in flow and it facilitates focused attention, which in turn facilitates engagement, which facilitates time distortion, which leads to enjoyment [64]. Researchers have found that the greater the perceived challenge, the greater the focused attention as long as the challenge is not so great that it creates anxiety [49].

### 7.2. Brain Synchronization for Attention Also Enhances Enjoyment and Connection

Group physiological synchrony was related to improvement in the emotional climate of the classroom [4]. Practices related to brain synchrony have been addressed here with regard to attention. However, it also enhances positive emotions—heart engagement. Brain synchrony is enhanced through shared emotions [129], tying the brain to the heart. Brain synchronization across individuals via emotions can promote social interaction [129], increasing connection. The synchronization of emotions can help individuals “get on the same wavelength” or “get in synch” [223]. The closer the student felt to the teacher, the less effect the teaching style (video vs. lecture) had on synchrony [12,15], and higher synchrony between students was related to higher reported social closeness to other students. [15].

### 7.3. Heart Synchronization for Enjoyment and Connection Also Enhances Engagement

I addressed practices from heart synchronization to enhance connection and enjoyment and practices related to flow to increase engagement. However, heart synchrony also increases engagement [15], another component in our ideal learning environment. Early studies of engagement focused on cognitive measures overlooking the important emotional component of engagement [16]. Emotional states affect engagement, and these states can be used to determine a learner’s engagement [16] Chaouachi and Frasson found that engagement was higher when the learners’ emotional state was positive with high arousal.

Secondarily, engagement was high when emotions reflected confusion or frustration, probably reflecting that the learner is engaged in trying to figure something out, which as we have seen in the discussion of flow, is a positive experience if the challenge is not so difficult that the frustration reaches the point of disengagement. Therefore, a little stress can assist in creating flow [224]. Conversely, too many right answers lead to reduced challenge and boredom, reducing engagement.

### 7.4. Heart Synchronization for Enjoyment Enhances Attention and Connection

We have seen how attention can be increased through engagement facilitated by flow and through joint attention leading to brain synchronization. Heart synchrony can also reflect shared attention [109,225]. In addition, heart synchrony is seen as indicative of connectedness or shared cognitive process, and is seen in research on collaborative learning [104]. Rein and McCraty‘s research implies that coherent heart energy can mediate heartfelt communication between individuals [226]. When individuals act in unison, heart rate synchrony increases [227]. We can use heart synchrony to attain the shared attention state, which can lead to brain synchrony.

Furthermore, emotional stimuli can increase attention unconsciously in a bottom-up process [109,228,229]. Positive emotions build competency and widen the focus of attention [230,231] and are associated with better achievement [3]. The process can be broken down to say emotion drives attention and attention drives learning [228,232]. Mcraty and Atkinson [165] found that feelings of gratitude created physiological coherence—a state of stability and efficiency in the heart, respiratory, blood pressure, and brain rhythms. This was dose-responsive: the more gratitude, the greater the coherence. This strengthened the neural pathways in the brain, firing and wiring coherence pathways. The researchers conclude that gratitude is a way of achieving flow. Focused attention is essential to get into a flow state [49,60,233,234,235]. This brings us full circle.

While we always strive to improve our teaching methods, it is also important to change our perspective on the background, the big picture, the gestalt: the classroom psychological and physiological environment. The emphasis currently is predominantly on content and practices or, sometimes, on the emotional state of individuals, rather than the mental/emotional environment in which these practices take place and in which a student must regulate emotions and perform academically. Even the current movement of teaching social/emotional skills has more of a focus on specific behaviors and interactions rather than the gestalt, which includes content and emotion—mind, brain, and heart. Even if the educator reading this just walks away with an awareness that the “whole” is to be considered, not just the collection of “parts”, that is a start. We cannot fix something we are not aware of. Once educators momentarily change their focus from the “face” to the “vase”, they see things differently. They can begin to make small changes toward a bigger goal. This changes the focus somewhat and changes how they do what they do. They can begin easily with a gratitude practice and work from there. They take less than five minutes to instill positive emotion by activating appreciation, compassion, and gratitude, thus getting a much greater return on the remaining time spent, and ultimately creating the ideal learning environment through individualized or group tasks that create flow, require joint attention either as a class or within groups, and engage the heart. Set a climate of engagement, attention, connection, and enjoyment as the North Star and work toward it. Then, when practices and environment are in place, we truly have a connected classroom that is “in the Synergy Zone”.

**Figure 1:**
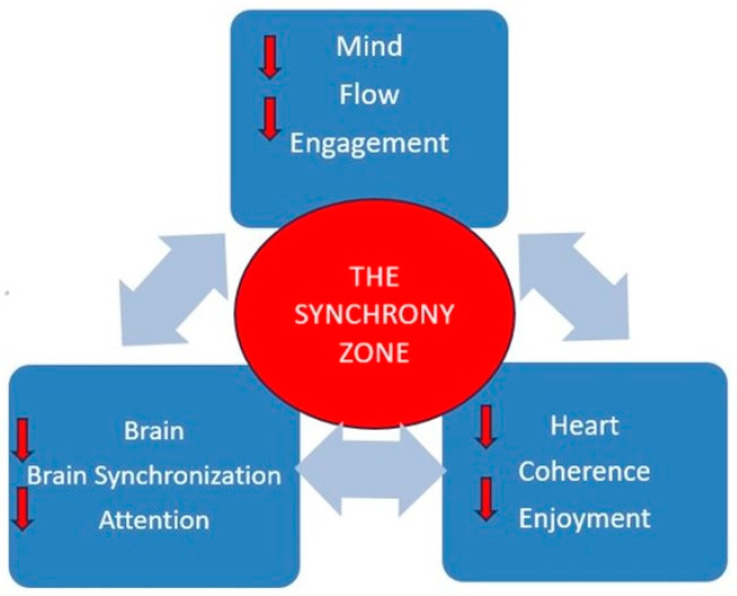
The Synergy Zone.

Figure 1 shows how all three aspects influence each other as indicated by the larger arrows pointing to the blue boxes. Within the blue boxes, you can see the top-down influences, i.e., the mind can create flow and flow can create engagement. When all three blue boxes are “activated”, the classroom could be in The Synergy Zone.
